# Mast Cells in Autism Spectrum Disorder—The Enigma to Be Solved?

**DOI:** 10.3390/ijms25052651

**Published:** 2024-02-24

**Authors:** Eleonora Kovacheva, Maria Gevezova, Michael Maes, Victoria Sarafian

**Affiliations:** 1 Department of Medical Biology, Medical University-Plovdiv, 4000 Plovdiv, Bulgaria; eleonora.kovacheva@mu-plovdiv.bg (E.K.); mariya.gevezova@mu-plovdiv.bg (M.G.); 2 Research Institute, Medical University-Plovdiv, 4000 Plovdiv, Bulgaria; dr.michaelmaes@hotmail.com; 3 Sichuan Provincial Center for Mental Health, Sichuan Provincial People’s Hospital, School of Medicine, University of Electronic Science and Technology of China, Chengdu 610072, China; 4 Key Laboratory of Psychosomatic Medicine, Chinese Academy of Medical Sciences, Chengdu 610072, China; 5 Department of Psychiatry, Faculty of Medicine, Chulalongkorn University, Bangkok 10330, Thailand; 6 Cognitive Fitness and Technology Research Unit, Faculty of Medicine, Chulalongkorn University, Bangkok 10330, Thailand; 7 Department of Psychiatry, Medical University-Plovdiv, 4000 Plovdiv, Bulgaria; 8 Kyung Hee University, 26 Kyungheedae-ro, Dongdaemun-gu, Seoul 02447, Republic of Korea

**Keywords:** ASD, MCs, neuroinflammation, cytokines

## Abstract

Autism Spectrum Disorder (ASD) is a disturbance of neurodevelopment with a complicated pathogenesis and unidentified etiology. Many children with ASD have a history of “allergic symptoms”, often in the absence of mast cell (MC)-positive tests. Activation of MCs by various stimuli may release molecules related to inflammation and neurotoxicity, contributing to the development of ASD. The aim of the present paper is to enrich the current knowledge on the relationship between MCs and ASD by discussing key molecules and immune pathways associated with MCs in the pathogenesis of autism. Cytokines, essential marker molecules for MC degranulation and therapeutic targets, are also highlighted. Understanding the relationship between ASD and the activation of MCs, as well as the involved molecules and interactions, are the main points contributing to solving the enigma. Key molecules, associated with MCs, may provide new insights to the discovery of drug targets for modeling inflammation in ASD.

## 1. Introduction

MCs are hemopoietic-derived tissue immune cells which are associated with allergies, but also participate in immunity [1] and inflammation [2]. They can produce mediators, both pro- and anti-inflammatory [3], and may have functions related to immune modulation [1,4]. In humans, two common MC phenotypes are recognized according to their protease content: MCs that contain only tryptase (MCTs) and MCs that contain both tryptase and chymase (MCTCs) [5]. MCTC predominate in the skin and connective tissue and are also present in significant numbers in the submucosal tissues of the respiratory tract. MCTs are more abundant in mucosal epithelium and in the lamina propria. Their roles are rather unclear, but their ability to release different proteases and cytokines suggests some mutually exclusive functions [5]. MCs function as protectors by responding to a wide range of “signals” (e.g., environmental antigens, allergens, invading pathogens or toxins) in a rapid and selective way [6]. For this purpose, they are equipped with a number of receptors, which induce the release of many biologically active products and may even lead to in situ proliferation of terminally differentiated MCs [4]. Furthermore, the activation of MCs by IgE-dependent triggers or other agonists is also related to significant alterations in the immunophenotypic profile of these cells. Some of the changes reflect MC degranulation, including the surface membrane expression of LAMP-1 (CD107a), LAMP-2 (CD107b) and LAMP-3 (CD63) [7].

ASD is a neurodevelopmental disease characterized by deficits in verbal and nonverbal communications, disturbance in social interactions and limited, repetitive patterns of behavior, interests and activities [8]. According to the Centers for Disease Control and Prevention, Autism and Developmental Disabilities Monitoring Network, approximately 1 in 36 children is identified with ASD (https://www.cdc.gov/ncbddd/autism/data.html accessed on 15 December 2023). Although some putative autism susceptibility genes have been identified [9] and gene interactions with environmental factors have been suspected, in the majority of cases, the cause of ASD remains unknown [10]. Some children with ASD regress at about age 3, often after a reaction to vaccination, infection [11], trauma [12], toxic exposures [13] or stress [14], implying the importance of environmental triggers [15].

Many children with ASD have a family or a personal history of “allergic symptoms”, often in the absence of positive skin or RAST (radioallergosorbent) tests. Furthermore, children with mastocytosis or MC activation syndrome (MCAS) develop ASD at a much higher rate than children of the general population. MC activation by allergic, infectious, environmental and stress-related stimuli, especially perinatally, would release molecules related to inflammation and neurotoxicity [16]. It has been suggested that mediators derived from MCs can alter the blood–brain barrier (BBB) and cause “brain allergy” [10] or “focal encephalitis” [17], thereby contributing to the pathogenesis of ASD [17,18]. Increased MC responsiveness may define at least one subset of patients with ASD who could benefit from inhibition of MC activation [16].

The aim of the present review is to summarize the current knowledge on the relationship between the activation of MCs, MC-derived molecules and mediators in the pathogenesis of ASD. Key molecules associated with MCs may provide new insights to the discovery of drug targets for modeling the inflammatory processes observed in ASD.

## 2. Functions of MCs 

MCs have immunomodulatory and physiological functions in the epithelium, endothelium, and nervous system. These cells act as guardians of the immune system and participate in many biological processes, as well as in the maintenance of homeostasis [19]. MCs are considered crucial for tissue function and integrity [20]. A number of MC mediators, including nerve growth factor (NGF), platelet-derived growth factor (PDGF), vascular endothelial growth factor (VEGF), and fibroblast growth factor-2 (FGF-2), and also histamine and tryptase, induce epithelial cell and fibroblast proliferation [21]. Furthermore, MCs are involved in all steps of tissue repair, from the initial inflammatory reaction to extracellular matrix (ECM) remodeling [22]. Through the release of platelet-activating factor (PAF), leukotrienes, and the cytokines IL-1 and IL-8, MCs contribute to platelet activation and aggregation as well as to the extravascular deposition of fibrin [23,24]. Proteases, such as tryptase, signal nerves through protease-activated receptors (PARs) [25,26,27,28]. MCs can also be activated by substance P and endothelin-1 (ET-1) [29,30]. MC–neuron interactions also contribute to the maintenance of intestinal homeostasis by regulating ion transport, vascular permeability, the secretory activity of mucus-producing cells, and gastrointestinal motility [31]. Moreover, the role of MCs in angiogenesis is most certainly related to the release of a large spectrum of angiogenic mediators, which include angiopoietin-1, FGF-2, VEGF, IL-8, TGF-β, TNF-α, histamine, heparin, tryptase and chymase, among others [32]. Similar to dendritic cells, MCs are among the first cells of the immune system to interact with antigens, toxins, and pathogens. In addition, MCs express various surface receptors which are able to detect potentially harmful signals and enable a rapid and appropriate response through the release of pre-stored and newly synthesized mediators. MCs recognize pathogens through the direct binding of pathogens or their components to pathogen-associated molecular pattern (PAMP) receptors on an MC’s surface, binding to complement or immunoglobulin receptors, or the recognition of endogenous peptides produced by infected or injured cells [33]. In vitro studies have shown that MCs are also capable of processing and presenting antigens via MHC-I and MHC-II complexes [34,35,36]. MCs are recognized as the main effector cells responsible for IgE-mediated allergic reactions [37,38]. 

## 3. MCs and Inflammation

The activation of mammalian MCs is triggered by a variety of signals generated during innate responses. Such signals include elements of the complement system [39,40,41], agonists of Toll-like receptors (TLRs) [41,42,43], adenosine [44], corticotropin-releasing factor receptors [45], numerous endogenous peptides, including vasoactive intestinal peptide (VIP) [46,47], and substance P [46,48] and a large number of other stimuli [41]. Contrary to stimulating signals, the reported suppressing MC activation signals are quite limited. 

Increasing evidence indicates that MCs are critical for the pathogenesis of inflammatory diseases [49,50], such as arthritis [51], atopic dermatitis, psoriasis [52,53], and multiple sclerosis [54]. Gene array analysis of human MCs activated by IgE showed the overexpression of numerous, mostly inflammation-related genes [55]. Moreover, proteases released by MCs could act on plasma albumin to generate histamine-releasing peptides [56,57] that would further propagate MC activation and inflammation. Proteases could also stimulate PARs, inducing microleakage and widespread inflammation [58,59]. However, unlike allergic reactions, MCs are rarely seen to degranulate during inflammatory processes. The only way to explain MC involvement in non-allergic conditions would be through “differential” or “selective” secretion of mediators without degranulation [60]. 

## 4. MCs, Neuroinflammation and ASD

The localization of MCs and, more importantly, the mediators released by both MCs and neurons collaborate in the establishment of a neuroimmune interaction between these cells. It has been shown that communication between MCs and neurons can occur through synaptic-like structures sustained by adhesion molecules such as N-cadherin or the synaptic cell adhesion molecule SynCAM [61,62]. 

**Figure 1:**
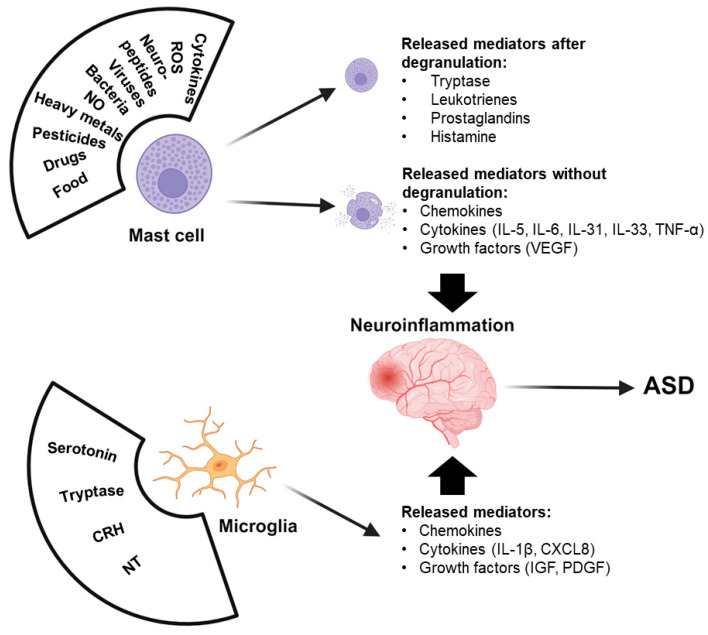
Schematic illustration of ASD pathogenesis from the perspective of MCs. Legend: CRH—Corticotropin-Releasing Hormone; NT—Neurotensin; ROS—Reactive Oxygen Species; NO—Nitric Oxide; IL—Interleukins; TNF-α—Tumor Necrosis Factor alpha; VEGF—Vascular Endothelial Growth Factor; IGF—Insulin-like Growth Factor; PDGF—Platelet-derived growth factor.

Microglia are specialized macrophages within the central nervous system (CNS) and they have an important role in neuroinflammation [63,64] and neurodegeneration [63,65]. Microglial activation is reported in ASD [66,67] and the release of pro-inflammatory mediators IL-1β and CXCL8 [68] (Figure 1). The transition of microglia from a resting state to an activated pro-inflammatory phase in ASD is regulated by different factors. Microglia can be activated by PAMPs and endogenous damage-associated molecular patterns (DAMPs), acting on TLRs, but also in response to histamine and tryptase released by MCs [69]. Triggered brain MCs have been shown to participate in cognitive dysfunction through microglial activation and neuronal apoptosis [70].

Macrophages are polarized due to changes in their environment, which result in different subtypes, such as M1 and M2 [71]. Lipopolysaccharides can drive macrophage polarization to the M1 phenotype, while IL-4 induces macrophage polarization to M2 [72]. M1 macrophages are related to pro-inflammatory responses and produce pro-inflammatory factors, whereas M2 macrophages are capable of anti-inflammatory responses and repair damaged tissues [73]. Based on the triggers and transcriptional changes, M2 macrophages are divided into four subgroups: alternative activated macrophages (M2a), type 2 macrophages (M2b), deactivated macrophages (M2c) and M2d [73]. M2a macrophages are involved in the clearance of apoptotic cells, modulating the inflammatory response and wound healing [74]. Both IL-4 and IL-13 are capable of inducing M2a polarization [75,76]. 

While IL-4 activates the IL-4Rα receptor, IL-13 triggers the IL-13Rα1 receptor. The two receptors can form heterodimers that respond to both IL-4 and IL-13 [75]. IL-5 activates the IL-5Rα receptor, which induces eosinophilia [75]. In case of exposure of M2A to IL-4 or IL-13, a loss of the specific M1 marker, inducible nitric oxide synthase (iNOS), follows [76]. Typical markers for M2A polarized macrophages and microglia are the arginase enzyme Arg1, scavenger receptors and the mannose receptor [76,77]. In addition, the transcription of growth factors such as VEGF, BDNF and PDGF is increased in IL-4-induced M2A microglia [76]. In the M2A-polarized state, microglial cells also express insulin-like growth factor-1 (IGF1) [77]. As a consequence, allergy may indirectly lead to the release of many potent growth factors from microglia. Upon IL-4 or IL-13 stimulation, the expression of IGF1 by microglia [77,78] and macrophages [78] is greatly increased. IGF1 is an important cytoprotective protein with a relevant function in tissue repair after inflammation [79]. Compared to controls, blood levels of IGF1 were significantly elevated in men with ASD who had a large head circumference. Moreover, head size was also found to correlate with IGF1 blood levels [80].

MCs interact with microglia in the brain [81] and their activation [82,83], especially in the hypothalamus [84], can lead to cognitive dysfunction [85]. Microglia express receptors for CRH [86] and can be further activated by stress [87]. Microglia are also rich in receptors for NT [88], which have been reported to be increased in the serum of patients with ASD [89] and can activate microglia to secrete pro-inflammatory molecules [68]. Microglia also express neurotensin receptor 3 (NTS3), the activation of which leads to microglial proliferation [90].

Increased gene expression of the pro-inflammatory microRNA-155 (miR-155) has been also found in the amygdala of children with ASD [91], as well as decreased expression of the anti-inflammatory cytokine IL-38 [92]. A transcriptome study of 104 human brain cortical tissue samples showed a relationship between the gene expression module of M2 microglia activation and a neuronal module, suggesting dysregulated microglial responses that may lead to alterations in neuronal activity in ASD [66].

## 5. MC Cytokines in ASD

The increased levels of proinflammatory cytokines [93,94,95,96,97,98,99] and the decrease in the anti-inflammatory cytokine IL-10 are some of the immune hallmarks of ASD [94,100,101,102,103]. This may be important as the balance of pro- and anti-inflammatory cytokines modulates brain development. Abnormal levels of these molecules are associated with a number of complex disorders, including ASD [101]. Generally, increased levels have been recorded for IL-5, IL-8, IL-13, IL-17 [102,104,105,106,107,108], IL-12 [109], IL-21, IL-22 [107], IFNγ [98,110], TNF-α [111], TNF-receptor II17, IL-1β, and IL-6. IL-1β is associated with impairments in memory and learning [112,113], and IL-6 is linked with stereotyped behavior, and impacts synapse formation [114,115,116]. These cytokines can modulate brain function by affecting cognitive and emotional processing, mood and sleep disorders in ASD [117]. 

TNF-α levels are positively related to the severity of ASD symptoms [118,119]. IL-1β has been found to increase the production of IL-17, which is a mediator of IL-8 [120]. Also, higher levels of IL-8 have been associated with more deviant behavior in patients with ASD, including hyperactivity, low language and cognitive ability [102]. Once activated, IL-8 attracts neutrophils to the areas of inflammation, leading to the release of proteolytic substances [121]. Cytokines secreted by immune cells can cross the blood–brain barrier and participate in the activation of microglia. Two recent studies demonstrate altered cytokine profiles in newborn infants, suggesting early immune dysregulation. Elevated IL-4 is associated with higher severity of ASD, while excessive IL-1β is linked to a milder form of the disease [122]. Increased levels of IFNγ, IL-4 and IL-5 in maternal serum are significantly associated with an increased ASD risk in the newborn [123]. 

MCs also secrete transforming growth factor-β (TGF-β), which promotes the development of Th17 cells and IL-17 production [124]. Elevated IL-17 has been reported in the serum and in immune cells of children with ASD [125]. MCs also release newly synthesized lipid-derived mediators such as prostaglandin D2 [126], cytokines (IL-5, IL-6, IL31, IL-33, and TNF) and chemokines (CCL5 and CXCL8) [127]. Furthermore, MCs can be influenced by cytokines, such as IL-1b. It induces the selective release of IL-6 [128] and IL-33 [129], which are enhancers of allergic MC stimulation [129,130]. IL-33 has also been shown to significantly increase the ability of substance P to stimulate MCs’ release of VEGF [131], TNF [132] and IL-1b [133]. 

A number of studies report the overproduction of proinflammatory cytokines in ASD children [100,118,134,135,136,137]. Significantly increased plasma levels of several cytokines, such as IL-1β, IL-6, IL-8, and IL-12p40, were found in ASD children compared to controls [118]. Another meta-analysis revealed significantly higher concentrations of IL-1β, IL-6, IL-8, interferon-gamma (IFN-γ), eotaxin, and monocyte chemoattractant protein-1 (MCP-1) in ASD individuals compared to controls [138]. Elevated proinflammatory cytokines were also found within the CNS (specifically in brain tissues and cerebrospinal fluid) of ASD patients [98]. On the other hand, the immune system also comprises regulatory elements that counter-regulate proinflammatory effects to maintain homeostasis, including anti-inflammatory cytokines, such as the IL-1 receptor antagonist (IL-1RA), IL-4, IL-10, and TGF-β [105,139,140]. However, studies regarding anti-inflammatory cytokines in ASD, as well as attention deficit hyperactivity disorder (ADHD), reflect inconsistent findings. While one meta-analysis reported significantly decreased levels of TGF-β1 and borderline increased levels of IL-1RA [138], a more recent meta-analysis revealed decreased levels of IL-10 and IL-1RA, and slightly increased levels of IL-5 in ASD patients [141]. 

## 6. The Role of MCs in ASD

Various perinatal allergic, genetic, environmental, immune and infectious factors may increase the risk of developing ASD [142,143]. They can activate MCs containing and releasing mediators, such as preformed kinins and proteases, and de novo synthesized leukotrienes, prostaglandins, chemokines (CCXL8, CCL2), cytokines (IL-4, IL-6, IL-1, TNF) and VEGF [144], as described in Figure 1. In addition, MCs function as an “immune gate to the brain” [145].

A study of 400 patients with mastocytosis revealed 15 cases with both mastocytosis and ASD—an incidence of 3.75 per 100, or 6.75 times higher than reported for the general population (1 in 180). In other families, mothers with mastocytosis have at least one child with ASD. As both ASD and mastocytosis are rare diseases, this association seems to be impressive [146]. Cerebro-spinal-fluid (CSF) levels of TNF-α were found to be significantly higher than the corresponding serum levels in ten children with autism [111], which may affect cognitive functions [147].

MCs are also stimulated by bacteria, viruses, fungi, drugs, foods, heavy metals, organophosphates and some neuropeptides, including CRH [148], neurotensin (NT) [149] and substance P [150]. Both NT [151] and substance P [152] are known to be involved in inflammatory processes. MC-derived mediators [2,127,144] can activate microglia [68,153] and cause localized inflammation [18,154,155], leading to ASD symptoms. NT is found to be elevated in young children with autism [156] and has been proposed as a possible therapeutic target due to its ability to induce neurotoxicity [157]. NT and CRH, secreted under stress, synergistically stimulate MCs and lead to increased vascular permeability [149] and blood-brain barrier (BBB) breakdown [158]. In addition, NT has been reported to stimulate MCs’ secretion of VEGF [159]. NT also increases the expression of CRH receptor 1 (CRHR-1) [160], whose activation by CRH enhances allergic stimulation of human MCs [161]. Furthermore, NT is elevated in the skin after acute stress, stimulates cutaneous MCs and increases vascular permeability in rodents [162]. NT triggers rodent peritoneal MCs to secrete histamine, which results in high plasma histamine levels by activating a specific NT receptor (NTR) [163]. Substance P induces the ST2 receptor for IL-33 [132], which further enhances MC activation through the combined action of neuropeptides and IL-33. Other neuropeptides, such as VIP and calcitonin gene-related peptide (CGRP) have been reported to be higher in the serum of children with ASD and in those with intellectual disability without ASD. In contrast, substance P and NGF levels were similar to controls [164]. Nevertheless, the same authors later found no differences in any of these peptides between autistic and control groups [165].

MCs are located perivascularly in all tissues, including the brain, where they are adjacent to CRH-positive nerve endings [166]. They are also plentiful in the meninges [167]. Studies have found that the stimulation of MCs leads to activation of microglia, which are actually involved in ASD [66] (Figure 1). In addition, MCs are also implicated in the regulation of the hypothalamus–pituitary–adrenal (HPA) axis in the skin [168] and brain [169], as histamine, IL-6 [170] and CRH [171] can activate this axis. MCs are usually stimulated by exposure to allergens and binding of IgE to high-affinity receptors (FcERIs), whose aggregation leads to degranulation and the secretion of multiple pre-stored and newly synthesized mediators [155,172]. In addition to IgE, many substances from the environment, gut or brain can induce MC activation [173]. MCs can serve as sensors of environmental and psychological stress [174], releasing danger signals [175] such as mitochondrial DNA (mtDNA) [160], which acts as an “innate pathogen” [176], causing autoinflammatory responses [177]. It is found to be increased also in the serum of children with ASD [178]. MCs can secrete exosomes that deliver miRNAs [179] and may be involved in brain pathology [180].

MCs may be implicated in ASD because of their unique ability to respond to non-IgE stimuli and release mediators “differentially” or “selectively” without the degranulation typical of allergic or anaphylactic reactions [60]. Bacteria and viruses have the capacity to trigger MCs via TLRs, which are important in the development of innate immunity. Lipopolysaccharides induce the selective release of TNF-α without degranulation via TLR-4, while peptidoglycans cause histamine release via TLR-2 from rodent MCs [181]. Human MCs express TLR-1, -3, -5, -7 and -9 [182].

The activation of TLR-9 selectively induces the production of the pro-inflammatory cytokine IL-6, while the activation of TLR-3 generates an IFN-α in response to double-stranded RNA [182]. These findings are important as neuroenteric viruses are likely to affect children at the age of 3–5 years [183] and contribute to immune abnormalities in ASD.

Environmental toxins such as mercury [184] and polychlorinated biphenyl (PCB) [185] have been related to developmental neurotoxicity [186] and ASD. Both mercury and PCBs can stimulate MCs. Furthermore, MCs can also be activated by aluminum [187]. Both mercury [188] and aluminum [189,190] have been associated with the severity of the symptoms in children with ASD [187]. In addition, herbicides such as glyphosate and atrazine have been reported to stimulate MCs and promote inflammation and neurotoxic effects [191] (Figure 1).

The risk of developing ASD may be further increased if children have mutations leading to decreased phosphatase and tensin homolog (PTEN). It is an inhibitor of the mammalian target of rapamycin, resulting in proliferation of microglia and MCs. Activation of susceptibility genes is often used to explain ASD. Stimulation of mTOR in patients with overactive mTOR as a result of gene mutations which lower PTEN would contribute to a form of “epigenetic” signal [192].

Several studies have found that atopic diseases [156,193] are associated with an increased risk of ASD [194], which is not surprising, as inflammatory processes, and interactions between stress and the immune system have been related to the pathogenesis of ASD [69,156]. A study of 1638 pregnant women stated that high levels of stress during gestation, and especially during the second trimester, are associated with high risk of ASD developing in the newborn at 6 months of age [195]. Psychological stress during pregnancy can cause immune system dysregulation and increased IgE levels in the umbilical cord blood [196]. This in turn may enhance the development of allergic reactions or sensitization to postnatal allergens in the fetus. In fact, children with ASD cannot handle stress [197] and have an exacerbated sense of fear [69], which can only worsen their condition. Elevated serum concentrations of IL-4 and IL-5 in women during the 15th and 19th weeks of gestation have been detected. These women gave birth to children who were later diagnosed with ASD [123]. Also, increased levels of IL-4, IL-10 and TNF-α have been measured in the amniotic fluid of fetuses that later developed ASD [198]. Another study noted that while IL-1β was associated with increased ratios of mild or moderate degree of ASD, high levels of IL-4 were linked with severe ASD. These findings show that peripheral cytokine profiles at birth are not only associated with ASD later in childhood, but also differ in terms of symptom severity [122]. Mothers who suffer from asthma, allergy, atopy or eczema during gestation are more prone to have children with a higher risk of neuropsychiatric problems [199]. Studies have reported strong a connection between ASD and food allergy [200] or food intolerance [201], which can lead to brain inflammation and cognitive impairment [202]. Msallam et al. [203] showed that circulating maternal immune IgE led to the vertical transmission of atopic dermatitis in the newborn by stimulating fetal MCs. In addition, both passive and active prenatal sensitization confer allergen sensitivity [203]. This indicates that fetal MCs are functional and can be stimulated by specific IgE and allergens coming from the mother during pregnancy. Although these studies are limited to lung and skin MCs, reactivity may also extend to brain MCs [204].

## 7. MCs and Therapeutic Targets

MCs, their triggers and their mediators could serve as unique therapeutic targets as they are influenced by the corticotropin-releasing factor (CRF) and appear to regulate the permeability of the gut–blood–brain barrier [158,169].

### 7.1. Serotonin Receptor Antagonists

In a study of six children with autism, four had high urinary serotonin levels [205]. Elevated platelet serotonin has been reported in more than 40% of autistic patients [206]. In a double-blind study of 40 ASD children randomized to the antipsychotic haloperidol and cyproheptadine versus haloperidol and placebo, the combined histamine H1 and serotonin receptor antagonist cyproheptadine was associated with significant clinical improvement [207].

### 7.2. MC Activation Blockers

Recent evidence suggests that MC activation is regulated by several costimulatory molecules [208]. Additional proof shows that MCs can be blocked through their inhibitory receptor FcgRIIb [209]. This inhibition, however, is relevant only for the allergic stimulation of MCs. Several MC mediators can inhibit their secretion too. Chondrotin sulfate, which is abundant in MC secretory granules, restrains mucosal MC activation [210]. The compound 48/80 induces histamine release, unlike disodium cromoglycate (cromolyn), which shows rapid tachyphylaxis [211].

### 7.3. Cromolyn

Cromolyn is a potent inhibitor of histamine secretion by rodent MCs, but a weak inhibitor of the allergic activation of human MCs [212]. Although it is poorly absorbed orally, it appears to reduce symptoms of mastocytosis, including neurobehavioral problems, indicating that gastrointestinal MCs affect the brain. The structure of cromolyn is similar to that of some flavonoids, polyphenolic compounds present in fruits, vegetables, nuts, seeds, and red wine with potent antioxidant, anti-inflammatory, and MC-inhibiting activities [213]. However, cromolyn does not effectively inhibit neither murine [214] nor human MCs [215,216,217]. What is more, it has been reported to potentiate histamine release from MCs [218].

### 7.4. Quercetin

Quercetin and other flavonoids inhibit the release of histamine, IL-6, IL-8, TNF-α, and tryptase from normal human MCs [219], making them possible candidates for the treatment of ASD. Given that MCs are activated by CRF, CRF receptor antagonists have been developed for several disorders [220] such as anxiety, neuroinflammation and irritable bowel syndrome [221], and may also be useful in ASD.

### 7.5. Rapamycin and Luteolin

Rapamycin and its analogues are mTOR inhibitors [222] which have been tested for the treatment of ASD [223,224,225,226]. Preliminary results show that the natural flavonoid luteolin is more potent than rapamycin in its ability to inhibit TNF release from human MCs [213]. A previous report also proved that the flavonoid-related epigallocatechin gallate (EGCG) is an mTOR inhibitor [227]. Luteolin may not only suppress mTOR, but also has additional beneficial effects on brain inflammation. It inhibits oxidative stress [213], inflammation [213], MC degranulation [228], MC cytokine release [161], thimerosal-induced inflammatory mediator release [229], microglial activation and proliferation [230,231,232], and autoimmune T-cell activation [233,234]. Luteolin also acts against methylmercury-induced mitochondrial damage [235], has a neuroprotective effect [236], and mimics BDNF [237,238]. Luteolin and quercetin also inhibit the release of histamine, IL-6, IL-8, TNF, and tryptase from human MCs [219,228]. Recently, tetramethoxyluteolin was found to be a more potent inhibitor of human MCs than luteolin [217]. In addition, luteolin has broad antiviral properties [239,240,241], and inhibits COVID-19 entry into host cells [242,243,244]. Furthermore, luteolin has better brain penetration and suppresses both microglia [68,245,246,247] and MCs [150,217]. It has a neuroprotective effect [248,249,250,251], and reduces neuroinflammation [249,252,253,254] and cognitive dysfunction [155,255,256,257], especially brain fog [258,259,260].

### 7.6. Extracellular Mitochondrial Components

It is revealed that the activation of MCs is accompanied by mitochondrial fission and translocation to the cell surface from where they secrete at least ATP and DNA outside the cell without any cell damage [160]. These extracellular mitochondrial components are misconstrued by the body as “innate pathogens” leading to powerful autocrine and paracrine auto-immune/auto-inflammatory responses. These mitochondrial constituents could be the “missing link” in certain auto-immune and auto-inflammatory diseases, especially in ASD [176]. Therefore, the clinical manifestation could be worse in the subgroup of ASD patients with mitochondrial dysfunction [261]. Preventing the secretion of extracellular mitochondrial components may be used as a novel therapeutic approach [176].

## 8. Conclusions and Perspectives

Data on the association of ASD with MCs are controversial and poorly understood. The involvement of MCs in innate and acquired immunity, gastrointestinal pathology, inflammation and increased intestinal permeability may explain the observed comorbidities in ASD. Patients with mutations in ASD susceptibility genes and hypersensitive MCs may represent a unique subgroup that are more likely to respond to environmental influences and stress. Activation of brain MCs by allergic, environmental, immune, neurohormonal, stress and toxic stimuli, particularly in domains related to behavior and language, can lead to focal brain allergies and focal encephalitis. Stress can worsen the clinical condition of ASD patients by stimulating MCs, leading to increased vascular permeability and neuroinflammation. Cytokines and key molecules, markers of MC degranulation, are shown to be implicated in ASD’s development.

Understanding the relationship between ASD, the activation and regulation of MCs, as well as the participating molecules and the interactions between them are the major points contributing to solving the enigma. They are important for the clarification of ASD pathogenesis and for the implementation of effective future treatments for ASD patients by providing novel therapeutic target molecules.
